# Reliable radiosynthesis of 4-[^10^B]borono-2-[^18^F]fluoro-l-phenylalanine with quality assurance for boron neutron capture therapy-oriented diagnosis

**DOI:** 10.1007/s12149-018-1268-6

**Published:** 2018-06-05

**Authors:** Kiichi Ishiwata, Ryoichi Ebinuma, Chuichi Watanabe, Kunpei Hayashi, Jun Toyohara

**Affiliations:** 1 Institute of Cyclotron and Drug Discovery Research, Southern TOHOKU Research Institute for Neuroscience, 7-115 Yatsuyamada, Koriyama, 963-8052 Japan; 2 Department of Biofunctional Imaging, Fukushima Medical University, Fukushima, Japan; 3 Research Team for Neuroimaging, Tokyo Metropolitan Institute of Gerontology, Tokyo, Japan; 4 Frontier Laboratories, Koriyama, Japan; 5 SHI Accelerator Service, Tokyo, Japan

**Keywords:** l-[^18^F]FBPA, [^18^F]F_2_ production, Quality control, PET, BNCT

## Abstract

**Objective:**

The aim of this study was to establish a reliable and routine method for the preparation of 4-[^10^B]borono-2-[^18^F]fluoro-l-phenylalanine (l-[^18^F]FBPA) for boron neutron capture therapy-oriented diagnosis using positron emission tomography.

**Methods:**

To produce l-[^18^F]FBPA by electrophilic fluorination of 4-[^10^B]borono-l-phenylalanine (l-BPA) with [^18^F]acetylhypofluorite ([^18^F]AcOF) via [^18^F]F_2_ derived from the ^20^Ne(d,α)^18^F nuclear reaction, several preparation parameters and characteristics of l-[^18^F]FBPA were investigated, including: pre-irradiation for [^18^F]F_2_ production, the carrier F_2_ content in the Ne target, l-BPA-to-F_2_ ratios, separation with high-performance liquid chromatography (HPLC) using 10 different eluents, enantiomeric purity, and residual trifluoroacetic acid used as the reaction solvent by gas chromatography-mass spectrometry.

**Results:**

The activity yields and molar activities of l-[^18^F]FBPA (*n* = 38) were 1200 ± 160 MBq and 46–113 GBq/mmol, respectively, after deuteron-irradiation for 2 h. Two 5 min pre-irradiations prior to [^18^F]F_2_ production for ^18^F-labeling were preferable. For l-[^18^F]FBPA synthesis, 0.15–0.2% of carrier F_2_ in Ne and l-BPA-to-F_2_ ratios > 2 were preferable. HPLC separations with five of the 10 eluents provided injectable l-[^18^F]FBPA without any further formulation processing, which resulted in a synthesis time of 32 min. Among the five eluents, 1 mM phosphate-buffered saline was the eluent of choice. The l-[^18^F]FBPA injection was sterile and pyrogen-free, and contained very small amounts of D-enantiomer (< 0.1% of l-[^18^F]FBPA), l-BPA (< 1% of l-FBPA), and trifluoroacetic acid (< 0.5 ppm).

**Conclusions:**

l-[^18^F]FBPA injection was reliably prepared by the electrophilic fluorination of l-BPA with [^18^F]AcOF followed by HPLC separation with 1 mM phosphate-buffered saline.

## Introduction

4-[^10^B]borono-2-[^18^F]fluoro-l-phenylalanine (l-[^18^F]FBPA) was developed in 1991 as a probe for positron emission tomography (PET) to evaluate in vivo 4-[^10^B]borono-l-phenylalanine (l-BPA) used in boron neutron capture therapy (BNCT) for patients with malignant tumors [1]. Based on several basic studies that verified the usefulness of l-[^18^F]FBPA [2–5], l-[^18^F]FBPA PET has been clinically applied for this purpose [6–9] and expanded in limited numbers of PET facilities over the past 20 years [10–13], mainly because BNCT is performed using a nuclear reactor for neutron irradiation. Recently, a cyclotron that acts as an epithermal-neutron source for BNCT and that can be installed in the hospitals has been developed [14, 15]. Phase I and II clinical trials of l-BPA BNCT using this cyclotron are in progress at two institutes in Japan, including the Southern Tohoku BNCT Research Center at the Southern TOHOKU Research Institute for Neuroscience. Therefore, the importance of l-[^18^F]FBPA PET is increasing, and further basic studies on the characterization of l-[^18^F]FBPA have been reported in recent years [15–23].

l-[^18^F]FBPA has been synthesized by the electrophilic fluorination of l-BPA with carrier-added [^18^F]F_2_ or [^18^F]acetylhypofluorite ([^18^F]AcOF) produced by three different routes. [^18^F]F_2_ was originally produced by deuteron irradiation of carrier F_2_-containing Ne, termed the ^20^Ne(d,α)^18^F nuclear reaction [1]. However, the activity yields of [^18^F]F_2_ and the resultant l-[^18^F]FBPA were low. For example, Wang et al. prepared 444–518 MBq of l-[^18^F]FBPA from 5.55 GBq of [^18^F]F_2_ after deuteron irradiation for 2 h [24]. The molar activity of l-[^18^F]FBPA was also low because of carrier F_2_: 30–60 MBq/µmol [1]. The second and third routes used the ^18^O(p,n)^18^F nuclear reaction for carrier-added [^18^F]F_2_ production. In the former, [^18^F]F_2_ was produced by proton irradiation of highly enriched [^18^O]O_2_ gas followed by a second proton irradiation step for the release of [^18^F]F_2_ [25, 26]. In the latter, [^18^F]fluoride produced by proton irradiation of [^18^O]H_2_O was converted to [^18^F]F_2_ via [^18^F]fluoromethane [27]. The ^18^O(p,n)^18^F reaction can produce potentially large amounts of ^18^F compared with the ^20^Ne(d,α)^18^F reaction; therefore, the activity yields of l-[^18^F]FBPA synthesized via the second route (2 GBq [25] to 5.3 GBq [26]), and the molar activity (257 MBq/µmol [26]) has been improved. The third route was especially devised to give less carrier-added [^18^F]F_2_. Consequently, the molar activity of l-[^18^F]FBPA was the highest (3700 MBq/µmol), but the activity yields have not been clearly described [28].

In the future, the synthesis of l-[^18^F]FBPA by nucleophilic fluorination using no-carrier-added [^18^F]fluoride will be developed to obtain higher activity yields and higher molar activities of l-[^18^F]FBPA, as the synthesis of 2-deoxy-2-[^18^F]fluoro-d-glucose has progressed from the method using [^18^F]F_2_ to that using no-carrier-added [^18^F]fluoride. However, at present such radiosynthesis is still under development, although a preliminary synthesis was reported recently [29].

At the present stage of l-[^18^F]FBPA PET for l-BPA BNCT-oriented diagnosis, l-[^18^F]FBPA PET is applied to only a few patients per l-[^18^F]FBPA preparation and not to mass screening; therefore, a steady and reliable synthesis of l-[^18^F]FBPA is required. The molar activity of l-[^18^F]FBPA is not a critical issue. Among the three methods of l-[^18^F]FBPA synthesis described previously, Ne-derived [^18^F]F_2_ is produced simply and cost-effectively compared with ^18^O-derived [^18^F]F_2_. Therefore, the original method of l-[^18^F]FBPA synthesis has been adapted clinically to date; however, detailed procedures, the optimization of each process, and technical knowhow have not been described sufficiently in previous reports on the three methods [1, 8, 24–26, 28]. In the present study, we aimed to elaborate on the original method from the viewpoint of routine clinical use.

For this purpose, steady production of [^18^F]F_2_ is first essential. It is well known empirically that a short pre-irradiation step is essential before the main irradiation for ^18^F-labeling; however, systematic studies on [^18^F]F_2_ production have not been reported. A larger amount of carrier F_2_ in Ne target, for example 0.5% F_2_, has a benefit for the steady production of [^18^F]F_2_ but provides low molar activity, and the stoichiometric relationship in the fluorination of l-BPA with [^18^F]F_2_/[^18^F]AcOF should be considered carefully. For electrophilic fluorination of l-BPA, the original method used [^18^F]AcOF due to its higher selectivity than [^18^F]F_2_ [1], whereas the second and third methods employed [^18^F]F_2_ probably to avoid activity loss of activity during the conversion process from [^18^F]F_2_ to [^18^F]AcOF [25–27]. Regarding the formulation of l-[^18^F]FBPA, the most popular preparation method is purification by high-performance liquid chromatography (HPLC) using a reversed-phase column with 0.1% aqueous AcOH as the mobile phase followed by evaporation of the l-[^18^F]FBPA fraction and re-dissolution in physiological saline [1]. To avoid this time-consuming evaporation process, Vähätalo et al. separated l-[^18^F]FBPA by HPLC using physiological saline containing 1–2% EtOH and 0.01% AcOH as the eluent, and the l-[^18^F]FBPA fraction was used directly for injection in clinical studies [27]; however, the pH of this injection was not described, although it appeared to be below 4. Prior to this, Ishiwata et al. proposed HPLC separation with physiological saline alone without clinical use [30].

In the present study, we investigated (1) the importance of pre-irradiation for [^18^F]F_2_ production with an appropriate F_2_ carrier, (2) steady production of l-[^18^F]FBPA in relation to F_2_ content and l-BPA, (3) HPLC separation methods to provide injectable l-[^18^F]FBPA without any further formulation processing, (4) the optical purity of l-[^18^F]FBPA, and (5) analysis of residual trifluoroacetic acid (TFA) used as a solvent in radiosynthetic preparation of l-[^18^F]FBPA. To the best of our knowledge, no report on points (4) and (5) has been published previously. Findings for the three other points would also provide useful information for the radiosynthesis of l-[^18^F]FBPA using ^18^O-derived [^18^F]F_2_.

Labeled compounds and related terms are expressed according to the International Consensus Radiochemistry Nomenclature Guidelines recently recommended by an international Working Group on ‘Nomenclature in Radiopharmaceutical Chemistry and related areas’ [31, 32].

## Materials and methods

l-BPA was purchased from Sigma-Aldrich Chemical (St Louis, MO). l-BPA, l-FBPA, and d-FBPA were kindly supplied by Stella Pharma (Osaka, Japan). 2-, 3-, and 4-fluoro-d,l-phenylalanine (2-, 3-, and 4-FPhe, respectively) were purchased from Tokyo Chemical Industry (Tokyo, Japan). Normal saline (500 and 1000 mL plastic bag), distilled water (20 ml plastic ampule and 500 ml plastic bottle) for injection, and sodium phosphate corrective injection 0.5 mmol/ml (pH 6.5, 20 ml plastic ampule) were purchased from Otsuka Pharmaceutical (Tokyo, Japan). Other chemical reagents were obtained from commercial sources.

### Production of [^18^F]F_2_

An 18 MeV cyclotron (CYPRIS HM-18, 18 MeV protons and 9 MeV deuterons, Sumitomo Heavy Industries, Tokyo, Japan) was employed. Elemental [^18^F]F_2_ was produced via the ^20^Ne(d,α)^18^F reaction in Ne containing F_2_ in a cylindrical target chamber [30 mm inner diameter (i.d.) and 242 mm length] made of aluminum. The incident deuteron energy was 7.9 MeV. All deuteron irradiation processes were performed at a fixed current of 20 µA.

### Conditioning production of [^18^F]F_2_

To determine a suitable pre-irradiation protocol before the main [^18^F]F_2_ production for ^18^F-labeling, three or four successive 5 min irradiations were conducted within 1–39 day intervals. The content of F_2_ in Ne was set in the same range in each experiment: 0.1% (v/v) (*n* = 7), 0.15% (*n* = 7), and 0.2% (*n* = 5) by mixing 5% F_2_-containing Ne and pure Ne gases. The final pressure was set at 380 kPa. However, the actual F_2_ concentrations calculated from the pressure were determined to have certain ranges. After the end of the 5 min irradiation, the [^18^F]F_2_ produced was recovered with a maximum flow rate by the target pressure and absorbed into a tandem column of soda lime (No.1, Wako Pure Chemicals, Osaka, Japan, 9 mm i.d. × 80 mm length) and activated charcoal (Granular, Wako Pure Chemicals, 9 mm i.d. × 80 mm length). The average flow rates from maximum pressure (ca. 380 kPa) to 100 kPa were in range of 756–845 ml/min. The activity absorbed in the tandem columns was estimated as the total activity recovered from [^18^F]F_2_ production, and corrected for decay to the end of cyclotron bombardment (EOB).

### Synthesis of l-[^18^F]FBPA

l-[^18^F]FBPA (total 38 runs) was prepared by electrophilic fluorination with [^18^F]AcOF using a multipurpose synthesizer (CFN-MPS200, Sumitomo Heavy Industries) using a method that was a slightly modified method from previous reports [33, 34]. Two conditioning 5 min irradiations were performed, and a main irradiation for ^18^F-labeling was performed for 90–156 min (123 ± 17 min). In all three irradiations, the content of F_2_ in Ne was set at the same percentage of 0.10–0.30% (30–89 µmol: 0.10%, *n* = 3; 0.15%, *n* = 8; 0.20%, *n* = 23; 0.25%, *n* = 2; and 0.30%, *n* = 2). The [^18^F]F_2_ produced was passed through a column containing sodium acetate trihydrate or sodium acetate anhydrous (4 mm i.d. × 40 mm length), and the resultant [^18^F]AcOF was bubbled into 4 ml TFA containing l-BPA at room temperature with maximal flow rates: 426 ± 68 ml/min from the target pressure (ca. 380 kPa) to 100 kPa. To examine the effect of the l-BPA-to-F_2_ ratios on l-[^18^F]FBPA synthesis, the amount of l-BPA was varied in the range 14.2–33.3 mg (68–160 µmol: 14.2–14.8 mg, *n* = 2; 18.5 mg, *n* = 1; 25.1–25.3 mg, *n* = 3; and 29.3–33.3 mg, *n* = 32). The total (100%) recovered from [^18^F]F_2_ production was estimated as the summed activities of the sodium acetate column and the TFA solution. The radioactivity sensor using to monitor a reaction vial containing the TFA solution was calibrated according to the standard ^18^F-activity measured with a dose calibrator (CRC-15 PET, Capintec, Florham Park, NJ, USA).

The TFA solution was heated to 120 °C, and the TFA was removed using a 200 ml/min N_2_ flow. The residue was dissolved in 2 ml of the eluent used for preparative HPLC (described below). The solution was applied to HPLC separation, and the fraction with l-[^18^F]FBPA was obtained. The volumes of the l-[^18^F]FBPA fractions were estimated by weight (1.0 g = 1.0 ml), and the pH was measured using a pH meter (Laqua act, Horiba Scientific, Tokyo, Japan). In four of 38 runs, the l-[^18^F]FBPA fraction was collected through a 0.22 µm membrane filter (SLGVJ33RS, Merck Millipore, Darmstadt, Germany) for clinical purposes, and the sterility and apyrogenicity were examined. Filter integrity (> 150 kPa) was evaluated using a Millex/Sterivex integrity tester (Merck Millipore). The activity yields of l-[^18^F]FBPA at the end of synthesis (EOS) were normalized with respect to those produced by irradiation for 120 min.

### HPLC separation of l-[^18^F]FBPA

The column used for HPLC separation of l-[^18^F]FBPA was YMC-Pack ODS-A (S-5 µm, 20 nm, 20 mm i.d. × 150 mm length, YMC, Kyoto, Japan) with a guard cartridge ODS-A (S-5 µm, 12 nm, 20 mm i.d. × 10 mm, YMC). The 10 different mobile phases investigated are summarized in Table 1 (eluents **1**–**10**). Eluents of 10 and 5 mM phosphate-buffered saline (PBS) were prepared by mixing normal saline, sodium phosphate corrective injection 0.5 mmol/ml (pH 6.5), and distilled water to an isotonic ion strength of 0.15 mEq/ml. Eluent containing 1 mM PBS was prepared by adding 1/500 volume of sodium phosphate corrective injection 0.5 mmol/ml (pH 6.5) into normal saline. The flow rate was 10 ml/min, and the elution profile was monitored using an ultraviolet (UV, 260 nm) detector (UV 2715 Plus, Jasco, Tokyo Japan) and a radioactivity monitor (UG-PD1A, Universal Giken, Odawara, Japan). First, in the separation with eluent **1**, based on previous reports [1, 27], a major radioactive peak, and later other minor radioactive peaks and shoulder components were fractionated, l-[^18^F]FBPA and three by-products of 2-, 3-, and 4-[^18^F]fluoro-l-phenylalanine were identified by comparison of their retention times with those of the authentic compounds (enantiomeric mixtures in the case of fluorophenylalanines) in the HPLC analysis described below.

**Table 1:** Characteristics of 4-[^10^B]borono-2-[^18^F]fluoro-l-phenylalanine (l-[^18^F]FBPA) preparations separated by high-performance liquid chromatography using 10 different eluents

Eluent	*n*	Volume	l-[^18^F]FBPA^a^		RCP^b^	l-BPA^c^	pH
		mL	GBq	GBq/mmol	%	%	
(**1**) 0.1% AcOH water	3	16.2 ± 1.8	1.21 ± 0.04	71.4 ± 7.2	97.2 ± 1.1	0.97 ± 0.36	3.3 ± 0.3
(**2**) 0.01% AcOH water	4	11.9 ± 1.2	1.06 ± 0.20	66.4 ± 6.4	98.5 ± 1.0	0.43 ± 0.17	3.9 ± 0.2
(**3**) Saline	2	22.3	1.32	66.4	98.7	0.97	6.0
(**4**) 0.01% AcOH saline	9	24.0 ± 3.0	1.18 ± 0.19	86.6 ± 23.8	98.9 ± 0.8	0.87 ± 0.71	3.8^d^
(**5**) 1% EtOH, 0.01% AcOH saline	1	20.3	1.35	50.9	98.7	2.08	3.8
(**6**) 0.01% AcOH, 5 mM phosphate-buffered saline	3	13.8 ± 2.2	0.95 ± 0.05	83.0 ± 24.9	99.6 ± 0.1	0.26 ± 0.23	6.1 ± 0.1
(**7**) 0.01% AcOH, 1 mM phosphate-buffered saline	3	15.3 ± 0.7	1.22 ± 0.17	60.1 ± 12.3	99.5 ± 0.2	0.72 ± 0.40	4.4 ± 0.1
(**8**) 10 mM phosphate-buffered saline	2	14.1	1.51	72.5	97.0	0.55	6.8
(**9**) 5 mM phosphate-buffered saline	3	15.8 ± 1.3	1.14 ± 0.04	71.7 ± 1.3	98.0 ± 0.9	0.32 ± 0.06	6.8 ± 0.0
(**10**) 1 mM phosphate-buffered saline	8	13.2 ± 3.3	1.12 ± 0.14	92.8 ± 31.4	98.0 ± 0.5	0.41 ± 0.23	6.7 ± 0.0

Data are average ± standard deviation

^a^l-[^18^F]FBPA obtained at the end of synthesis was normalized to that produced by 120-min irradiation

^b^Radiochemical purity (RCP) was determined based on HPLC analysis

^c^Contamination (moles) of 4-[^10^B]borono-l-phenylalanine (l-BPA) is expressed as a percentage against the mass of l-FBPA.

^d^*n* = 2

### HPLC analysis of l-[^18^F]FBPA

The column used was YMC-UltraHT Pro C18 S-2 µm (3.0 mm i.d. × 100 mm length, YMC). Four different mobile phases were investigated: (a) 50 mM AcOH/50 mM AcONH_4_ (1/1), (b) 50 mM NaH_2_PO_4_, (c) 0.1% AcOH, and (d) 0.8% AcOH containing 1 mM ethylenediaminetetraacetic acid (EDTA) and 1 mM sodium octylsulfate [similar eluents as described in Refs. 1, 23, 25] at a flow rate of 0.5 ml/min at 20–21 °C, and the elution profiled was monitored using a UV detector at 280 nm (SPD-20A Prominence UV/VIS detector, Shimadzu, Tokyo, Japan) and a radioactivity monitor (US-3000, Universal Giken). The retention times of l-BPA and l-FBPA were 6.0 and 8.5 min, 6.1 and 8.7 min, 5.5 and 7.7 min, and 4.4 and 6.1 min with eluents **a, b, c**, and **d**, respectively. The retention times of 2-, 3-, and 4-FPhe were 8.5, 12.5, and 12.7 min, and 12.5, 14.1, and 19.9 min with eluents **a** and **d**, respectively.

### Optical purity of l-[^18^F]FBPA

A Crownpak CR (+) (4.0 mm i.d. × 150 mm length, Daicel, Tokyo, Japan) column was used with a mobile phase of HClO_4_ (pH 2.0) at a flow rate of 1.0 ml/min at 20–21 °C. The retention times of d-FBPA and l-FBPA were 6.5 and 9.9 min, respectively.

### Measurement of TFA in l-[^18^F]FBPA

Gas chromatography-mass spectrometry (GC-MS) was applied for the analysis of residual TFA in eight l-[^18^F]FBPA preparations: eluents **1**, *n* = 1; **4**, *n* = 5; **9**, *n* = 1; and **10**, *n* = 1. 0.1 ml of concentrated H_2_SO_4_/MeOH (4/1) was added to a 0.5 ml l-[^18^F]FBPA sample, and the mixture was shaken vigorously for 30 s. 0.4 ml of CH_2_Cl_2_ was then added to the mixture followed by a 15 s extraction with methyl trifluoroacetate. The CH_2_Cl_2_ phase solution (1 µl) was applied to GC–MS analysis 1 min after the end of extraction. Standard aqueous TFA (0.1–100 ppm) was also treated in the same way, and a calibration curve of methyl trifluoroacetate was prepared.

A quadruple mass spectrometer (5975C, Agilent Technologies, Santa Clara, CA) in conjunction with a gas chromatograph (5890GC, Agilent Technologies) was used with a deactivated metal capillary column (0.25 mm i.d. × 30 m) with 1 µm film thickness of Ultra ALLOY-CW (Frontier Laboratories, Koriyama, Japan). The oven temperature was maintained at 40 °C for 3 min and then increased to 150 °C at 100°C/min and held there for 3 min. The split/splitless injector and the transfer line were kept at 200 °C. The flow rate of He carrier gas was 1.5 ml/min.

## Results and discussion

### Conditioning production of [^18^F]F_2_

**Fig. 1:**
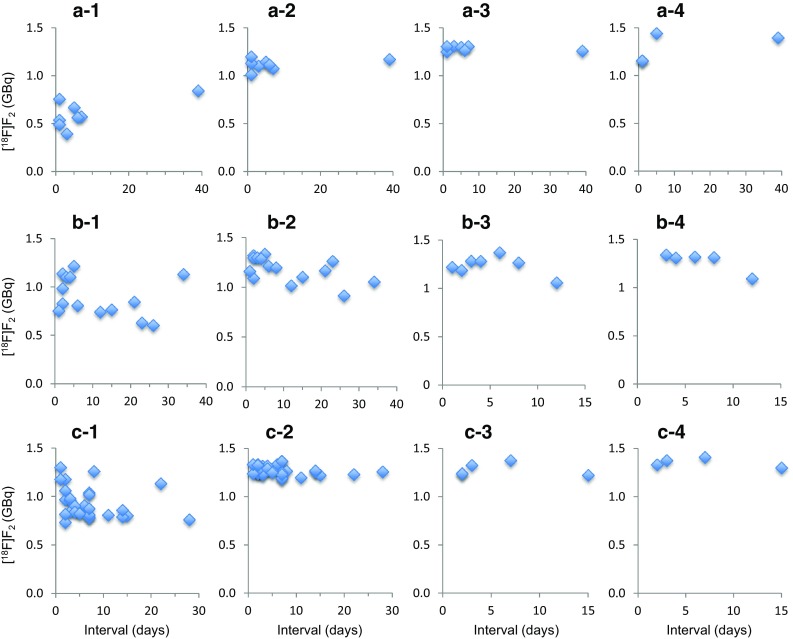
Effect of irradiation times and irradiation interval on the production of [^18^F]F_2_. **a**–**c** Show the activity yields (GBq) of [^18^F]F_2_ for 5 min irradiation of Ne containing 0.1%, 0.15%, and 0.2% F_2_, respectively, and –(**1**), -(**2**), -(**3**), and **(4**) indicate the first, second, third, and fourth 5 min irradiations, respectively

**Fig. 2:**
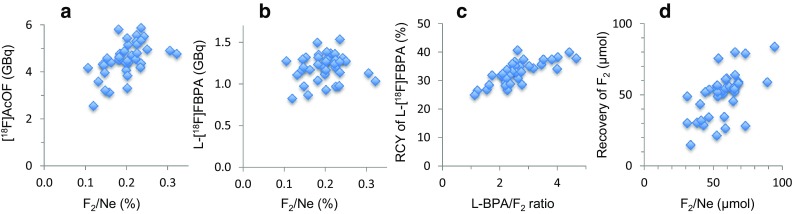
Effects of reaction conditions on the production of [^18^F]acetylhypofluorite ([^18^F]AcOF) and 4-[^10^B]borono-2-[^18^F]fluoro-l-phenylalanine (l-[^18^F]FBPA). **a** Relationship between F_2_ content (%) in Ne and activity yield of [^18^F]AcOF (GBq). **b** Relationship between F_2_ content (%) in Ne and activity yield of l-[^18^F]FBPA (GBq). **c** Relationship between 4-[^10^B]borono-l-phenylalanine (l-BPA)-to-F_2_ ratio (molar ratio) and radiochemical yield (RCY) of l-[^18^F]FBPA (%). **d** Relationship between F_2_ (mole) in Ne and F_2_ (mole) recovered as [^18^F]F_2_. The amount (mole) of [^18^F]F_2_ was calculated from the molar activity of l-[^18^F]FBPA and the total activity (summed activity absorbed in a sodium acetate column and recovered in a reaction vial as [^18^F]AcOF)

Figure 1 shows a plot of the activity yields of [^18^F]F_2_ after each of three or four successive 5 min irradiations in experiments for the conditioning production of [^18^F]F_2_ together with the yields in each of two conditioning 5 min irradiations for the l-[^18^F]FBPA synthesis as a function of the intervals of each irradiation day. The calculated actual F_2_ concentrations for 0.10%, 0.15%, and 0.20% F_2_/Ne targets were 0.11 ± 0.01% (range 0.08–0.14%, *n* = 27), 0.15 ± 0.02% (0.10–0.19%, *n* = 38), and 0.20 ± 0.02% (0.17–0.25%, *n* = 65), respectively. The activity yields were variable after the first irradiation regardless of the F_2_ content or interval days. For 0.10% and 0.15% F_2_/Ne targets, the third irradiations produced almost steady-state yields. With 0.20% F_2_/Ne, the yields reached steady state with the second irradiation. It is noted that steady-state yields are useful from a practical perspective but did not mean the quantitative recovery of [^18^F]F_2_ produced with each irradiation even if a higher concentration of F_2_ was employed. The relationship between the molar mass of F_2_ set and that of recovered is shown later (Fig. 2d).

In an early study on [^18^F]F_2_ production, a carrier concentration of 0.1% F_2_ produced almost 95% of the theoretical yield of [^18^F]F_2_, and a part of [^18^F]F_2_ was adsorbed on a stainless steel tube in recovery from the target chamber [35]. The present study demonstrated the clear requirement of pre-irradiation in a range of 0.1–0.2% F_2_, and suggested that two pre-conditioning irradiations with these F_2_ concentrations were preferable for steady [^18^F]F_2_ production. It was also suggested that mixing 5% F_2_-containg Ne and pure Ne gases made accurate setting of the F_2_ concentration difficult.

### Synthesis of l-[^18^F]FBPA using [^18^F]AcOF

The total activity of [^18^F]F_2_ (summed activities absorbed in a sodium acetate column and recovered in a reaction vial as [^18^F]AcOF) that was normalized as those produced by irradiation for 120 min, was 12.3 ± 1.9 GBq (*n* = 38). The activity yields of [^18^F]AcOF trapped in the reaction vial tended to increase with increasing the F_2_ percentage in Ne, and ≥ 0.15% F_2_ was preferable (Fig. 2a). The flow rates of [^18^F]F_2_ passing through a sodium acetate trihydrate column were slightly higher than those passing through a sodium acetate anhydrous column: 460 ± 89 ml/min (*n* = 23) vs. 363 ± 55 ml/min (*n* = 11, 4 data missing), and the respective radiochemical yields (RCYs) of [^18^F]AcOF were 38.8 ± 2.7% (*n* = 23) and 35.5 ± 1.9% (*n* = 11, 4 data missing) based on the total activity of [^18^F]F_2_ recovered. The difference in RCYs between the two cases was not large, but two other experiments using a sodium acetate anhydrous column with flow rates of 252 and 319 ml/min produced very low [^18^F]AcOF RCYs of 10.9 and 15.9%, respectively. These results suggested that low flow rates of [^18^F]F_2_ decreased the recovery of [^18^F]AcOF, and that sodium acetate trihydrate with larger particle sizes would be preferable compared with sodium acetate anhydrous with smaller particle sizes.

The activity yields of l-[^18^F]FBPA were variable and did not tend to increase with the F_2_ percentage in Ne (Fig. 2b). The averaged activity yield was 1200 ± 160 MBq (*n* = 38) at 31.6 ± 1.7 min from the EOB. The RCY of l-[^18^F]FBPA based on [^18^F]AcOF trapped in the reaction vial was 33.1 ± 3.8% (*n* = 38). The RCY increased with increasing l-BPA-to-F_2_ ratios (Fig. 2c), which suggested that the increased [^18^F]AcOF relative to l-BPA further fluorinated to produce ^18^F-difluorinated l-BPA as observed in the electrophilic fluorination of l-3-(hydroxy-4-pivaloyloxyphenyl)alanine with [^18^F]AcOF [36] and/or degraded l-[^18^F]FBPA. The preferred l-BPA-to-F_2_ ratio is > 2 for the steady production of l-[^18^F]FBPA. It is emphasized that the synthesis time (32 min) was the shortest compared with those in previous reports: 50 min [27], 72 min [26], 80 min [1], 88 min [25], and 110 min [24].

**Fig. 3:**
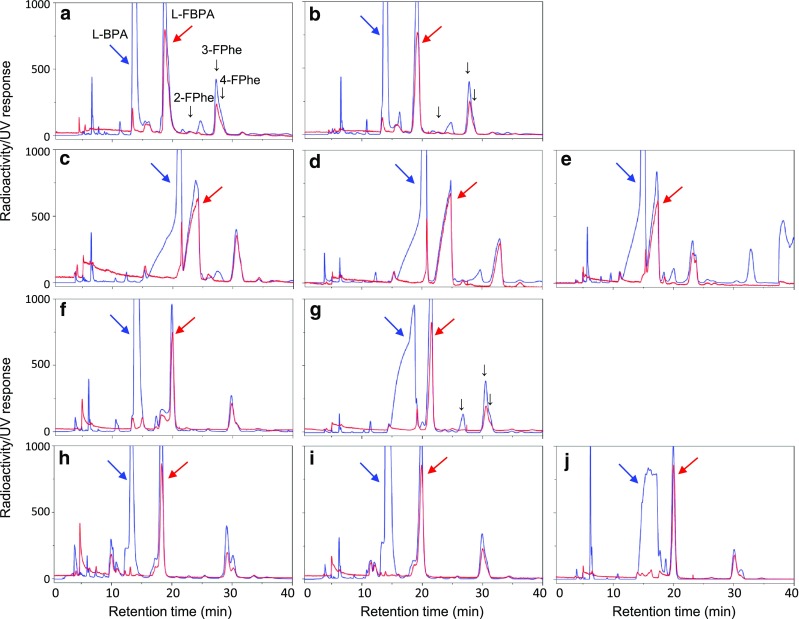
HPLC chromatograms of 4-[^10^B]borono-2-[^18^F]fluoro-l-phenylalanine (l-[^18^F]FBPA) separation using 10 different eluents. **a**–**j** Correspond to the chromatograms with eluents **1**–**10**. Red and blue lines show the elution profiles of radioactivity and ultraviolet (UV, 260 nm) absorbance, respectively. The unit of the vertical axis are the millvolt output of the UV detector. Elution positions of 4-[^10^B]borono-l-phenylalanine (l-BPA), l-FBPA, and 2-, 3-, and 4-fluoro-d,l-phenylalanine (2-, 3-, and 4-FPhe, respectively) are indicated

As previously described [1, 27], three by-products of 2-, 3-, and 4-[^18^F]fluoro-l-phenylalanine were tentatively identified by comparison with the retention times of authentic samples. Although baseline separation of 3- and 4-[^18^F]fluoro-l-phenylalanine could not be performed in all HPLC separations investigated, the relative amounts were in the order of 3- > 4- > 2-isomer (Fig. 3), and the summed RCYs of the three were constant at 11.6 ± 1.3% (*n* = 37). Electrophilic fluorination at the aromatic carbon 4 could explain undesired deboronation that leads to the 4-isomer; however, there is no known mechanism that produces the 2- and 3-isomers. Coenen et al. reported that the fluorination of l-phenylalanine in TFA with [^18^F]F_2_ produced 2- (72.5%), 3- (13.9%), and 4-[^18^F]fluoro-l-phenylalanine (13.6%) [37]. It is unlikely that l-phenylalanine produced after deboronation of l-BPA was fluorinated. We tried further identification of these byproducts by GC-MS as described for the determination of TFA using a deactivated metal capillary column (0.25 mm i.d. × 30 m) with 1 µm film thickness of Ultra ALLOY-1 (polydimethylsiloxane) (Frontier Laboratories), but could neither identify nor deny three byproducts to be 2-, 3-, and 4-[^18^F]fluoro-l-phenylalanine.

The molar activities of l-[^18^F]FBPA synthesized using 0.1%, 0.15%, 0.2% 0.25%, and 0.3% F_2_ were 103.5 ± 9.5 (*n* = 3), 86.1 ± 24.4 (*n* = 8), 69.0 ± 7.3 (*n* = 23), 66.0 (*n* = 2), and 50.3 GBq/mmol (*n* = 2), respectively. It is reasonable that lower carrier F_2_ contents in [^18^F]F_2_ production resulted in higher molar activities of l-[^18^F]FBPA; however, no linear relationship was found between the F_2_ content and molar activity. Figure 2d shows the molar amounts of recovered [^18^F]F_2_ that were calculated from the molar activity of l-[^18^F]FBPA and the total activity. Large differences observed between the amounts of F_2_ set in Ne and the recovered F_2_ indicated that the carrier F_2_ added could not be recovered constantly, even after two pre-conditioning irradiations.

Discussion of the differences between the present and previous studies on l-[^18^F]FBPA synthesis using Ne-derived [^18^F]AcOF is difficult because the detailed reaction conditions were not described in the previous reports. However, the present activity yields (1200 ± 160 MBq) were much higher than in previous reports: 444–518 MBq (*n* = 10) [24] and 750 ± 250 MBq (*n* = 8) [33 figures not shown], and the molar activities were higher than those in some reports [1, 23, 24] but lower than that reported in [9] (130 GBq/mmol).

Fig. 3 shows HPLC separation patterns with 10 different eluents. Eluent **1** 0.1% AcOH, a standard mobile phase used previously, gave a baseline separation (Fig. 3a); however, lower 0.01% AcOH (Fig. 3b) was preferable. Physiological saline (Fig. 3c) showed leading peaks of l-BPA and l-[^18^F]FBPA, but did not show baseline separation. The addition of AcOH to saline (Fig. 3d) improved the separation slightly. Further addition of EtOH (Fig. 3e) caused slightly faster elution but without improved separation. The addition of sodium phosphate corrective injection 0.5 mmol/ml (pH 6.5) to saline with/without AcOH improved the separation (Fig. 3f, h, i). Lower sodium phosphate eluted l-BPA more broadly, but l-[^18^F]FBPA was separated as an apparently single peak (Fig. 3g, j).

**Fig. 4:**
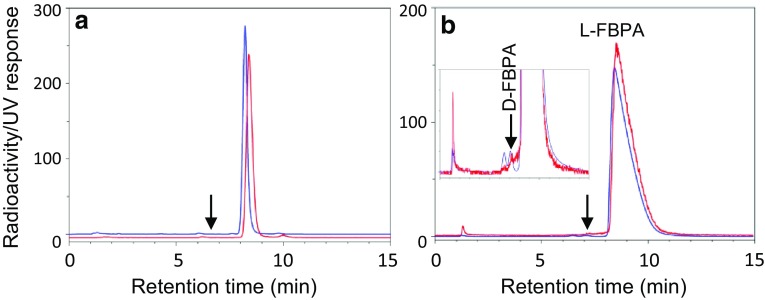
HPLC chromatograms of 4-[^10^B]borono-2-[^18^F]fluoro-l-phenylalanine (l-[^18^F]FBPA) analysis on **a** YMC-UltraHT Pro C18 S-2 µm and **b** Crownpak CR (+) columns. Red and blue lines show the elution profiles for radioactivity and ultraviolet (UV, 277 nm) absorbance, respectively. The units of the vertical axis are millivolt output of the UV detector. **a** In the stability test for 4 h, a radiochemical impurity appeared at the retention time indicated by the arrow. **b** The inset emphasizes the low levels of the chromatograms and the elution position of d-FBPA is indicated by the arrow

The characteristics of the 10 l-[^18^F]FBPA preparations are summarized in Table 1. First, the radiochemical purities (RCPs) of the four eluents for HPLC analysis were compared for five l-[^18^F]FBPA preparations. In analyses with eluents **(a**) 50 mM AcOH/50 mM AcONH_4_ (1/1) (Fig. 4a), **(b**) 50 mM NaH_2_PO_4_, **(c**) 0.1% AcOH, and **(d**) 0.8% AcOH containing 1 mM EDTA and 1 mM sodium octylsulfate, the RCPs were 97.7 ± 1.3%, 97.7 ± 1.4%, 98.6 ± 0.6%, and 98.4 ± 0.6%, respectively. Analyses with eluents **(a**) and **(b**) were similar, and preferable compared to eluents **(c**) and (**d**). Therefore, all subsequent analyses were conducted with eluent (**a**).

The RCPs were over 97% for all 10 l-[^18^F]FBPA preparations. The pH was below 4.0 in four of the 10 preparations. The volumes of l-[^18^F]FBPA separated with saline with/without 0.01% AcOH were over 20 ml, and the addition of sodium phosphate corrective injection reduced the volumes. Baseline separation could not be performed using eluents **3**–**5** and 1–2% contamination levels of l-BPA were obtained. Contamination levels of l-BPA in five other preparations with eluents **6**–**10** were very low. These five preparations were thus considered as acceptable for intravenous injection without any further processing. The l-[^18^F]FBPA eluted with 0.1% AcOH (eluent **1**) was previously used directly in a clinical study after the addition of 25% ascorbic acid injection and 10% sodium chloride injection to manage the pH and ion strength, respectively [33]. Comparison of activity yields and molar activities in the 10 preparations shown in Table 2 may not be significant, because the F_2_ contents were randomly set.

**Table 2:** Stability of 4-borono-2-[^18^F]fluoro-l-phenylalanine preparations separated by high-performance liquid chromatography using five different eluents

Eluent	*n*	pH	Radiochemical purity (%)^a^			
			0 h	1 h	2 h	4 h
(**3**) Saline	1	4.9	99.2	98.6	98.3	97.9
(**6**) 0.01% AcOH, 5 mM phosphate-buffered saline	2	6.1	99.6		97.9	97.6
(**7**) 0.01% AcOH, 1 mM phosphate-buffered saline	3	4.4 ± 0.1	99.5 ± 0.2	99.3 ± 0.4	99.0 ± 0.5	99.1 ± 0.8
(**9**) 5 mM phosphate-buffered saline	3	6.8 ± 0.0	98.0 ± 0.9		97.1 ± 1.6	96.7 ± 0.7
(**10**) 1 mM phosphate-buffered saline	4	6.7 ± 0.0	97.8 ± 0.9	97.4 ± 0.5	97.0 ± 0.5	96.7 ± 0.5

Data are average ± standard deviation

^a^The time for the first analysis after the end of synthesis was defined as “0 h”, and then analyses were performed successively at approximately the indicated intervals until approximately 4 h

From these results and the stability of l-[^18^F]FBPA described later, eluent **10** was selected for routine clinical use because of the small amount of one additive in physiological saline and ease of eluent preparation. The clinical injection volumes of l-[^18^F]FBPA separated with eluent **10** (1120 MBq/13.2 ml, Table 2), were expected to be 2.6–3.9 ml/60 kg when the radioactive injection doses in l-[^18^F]FBPA PET were 3.7–5.55 MBq/kg [38, 39], and one l-[^18^F]FBPA preparation can be used for 2 subjects with one PET scanner or 3–4 subjects with two PET scanners. In four l-[^18^F]FBPA preparations separated with eluent **10** and collected through a 0.22 µm membrane filter, sterility and apyrogenicity (< 0.0029 EU/ml) of the l-[^18^F]FBPA injection and filter integrity (≥ 330 kPa) were confirmed. It is noted that no radionuclidic impurities were found in l-[^18^F]FBPA prepared using the present method [34].

In previous research to improve the activity yields or the molar activity of l-[^18^F]FBPA, l-BPA was fluorinated with [^18^F]F_2_ produced by the ^18^O(p,n)^18^F reaction [25–27]. We also fluorinated l-BPA with [^18^F]F_2_ and separated l-[^18^F]FBPA by HPLC with eluents **1, 6**, and **8**; however, the RCPs (*n* = 6) were lower than those of [^18^F]AcOF-derived l-[^18^F]FBPA separated using the same eluents. The higher reactivity of [^18^F]F_2_ than [^18^F]AcOF may result in more side reactions. Therefore, no further investigation was conducted for the synthesis using [^18^F]F_2_.

### Stability

The stabilities of five l-[^18^F]FBPA preparations are summarized in Table 2. The l-[^18^F]FBPA separated by eluent **7** was the most stable over 4 h after EOS. In the four other preparations the RCPs decreased gradually but were maintained at over 96% for 4 h. Low pH with eluent **7** may contribute to the stability of l-[^18^F]FBPA; however, these five preparations without stabilizers such as EtOH [28] and ascorbate [33] were suitable for routine clinical use for at least 4 h. Preliminarily we found that the ascorbate was not effective for stability. In two l-[^18^F]FBPA preparations separated with 0.01% AcOH saline and 5 mM PBS, the addition of ascorbate injection (final concentration of 10 mg/ml) slightly decreased the RCPs from 98.4% and 97.7–93.2% and 91.3%, respectively, by approximately 4 h.

### Optical purity

No signal associated with the d-isomer was found in l-[^18^F]FBPA preparations, probably because of the low activity concentrations (Table 1, 47–107 MBq/mL) and the sensitivity of the US-3000 radioactivity detector. However, in the l-[^18^F]FBPA samples concentrated by evaporation or only the peak fraction of l-[^18^F]FBPA from HPLC separation (270–970 MBq/ml), a very small amount of the d-isomer was present (Fig. 4b): < 0.1% (0.09 ± 0.04%, *n* = 4) compared with the l-isomer. The UV peak that corresponds to this activity peak was increased by the addition of small amounts of standard d-FBPA into the samples. It was noted that a temperature at 20–21 °C was critical in this analysis because d-[^18^F]FBPA was not separated from the radioactive impurities at higher temperature (25 °C).

A UV peak corresponding to d-FBPA was evident and the UV detection sensitivity was higher than that of radioactivity. However, UV signals could not be used to evaluate d-[^18^F]FBPA, because the retention times of authentic d-FBPA and l-BPA coincided. No UV peak that corresponded to the d-isomer was observed for the starting compound l-BPA (both from Sigma-Aldrich and Stella Pharma); therefore, we considered that epimerization of l-[^18^F]FBPA might occur in TFA solution in the radiosynthesis process, as l-amino acids such as l-phenylalanine were epimerized in acetic acid [40].

### TFA analysis by GC-MS

The residual TFA in l-[^18^F]FBPA preparations was analyzed by GC-MS after methylation. The retention time of methyl trifluoroacetate in the GC stage was 2.5 min. Three ions were monitored in the SIM-EI^+^ mode of operation: *m*/*z* 59, 69, and 99. These are the most characteristic ions in the mass spectrum of methyl trifluoroacetate [41]. Because the signal at *m*/*z* 69 was much stronger than those at *m*/*z* 59 and 99; therefore, only the area of the *m*/*z* 69 peak was used for quantitative analysis. Methyl trifluoroacetate was degraded gradually by approximately 10% after 20 min from the end of extraction; therefore, the GC-MS analysis was started just 1 min after the end of extraction. The detection limit of methyl trifluoroacetate was determined to be 0.5 ppm [signal-to-nosise ratio (S/N) = 12], and the S/N of the 0.1 ppm standard sample was 8. In eight l-[^18^F]FBPA preparations the residual TFA was less than 0.5 ppm: 0.2 ± 0.1 ppm (range, 0.0–0.3 ppm) without evaporation processing for preparing the l-[^18^F]FBPA injection. It is noted that the LD_50_ values are 200 mg/kg in rats (oral administration) and 1200 mg/kg in mice (intravenous injection) (Hazardous Substances Data Bank, 2007: https://toxnet.nlm.nih.gov/cgi-bin/sis/search2/f?./temp/~TF1xcW:1).

## Conclusion

Two 5 min pre-irradiations enabled the steady production of [^18^F]F_2_ for ^18^F-labeling by electrophilic fluorination. To achieve a high RCY of l-[^18^F]FBPA 0.15–0.2% carrier F_2_ in Ne and an l-BPA-to-F_2_ ratio > 2 were preferable. HPLC separation using 1 mM PBS provided injectable l-[^18^F]FBPA without any further formulation processing, which resulted in a 32-min synthesis period from EOB. The l-[^18^F]FBPA injection contained small amounts of d-enantiomer (< 0.1% of l-[^18^F]FBPA), l-BPA (< 1% of l-FBPA), and TFA (< 0.5 ppm).
